# Risk of Re-Rupture, Vasospasm, or Re-Stroke after Clipping or Coiling of Ruptured Intracranial Aneurysms: Long-Term Follow-Up with a Propensity Score-Matched, Population-Based Cohort Study

**DOI:** 10.3390/jpm11111209

**Published:** 2021-11-16

**Authors:** Jiaqiang Zhang, Yang-Lan Lo, Ming-Chang Li, Ying-Hui Yu, Szu-Yuan Wu

**Affiliations:** 1 Department of Anesthesiology and Perioperative Medicine, People’s Hospital of Zhengzhou University, Henan Provincial People’s Hospital, Zhengzhou 450052, China; jiaqiang197628@163.com; 2 Department of Neurosurgery, Lo-Hsu Medical Foundation, Lotung Poh-Ai Hospital, Yilan 26546, Taiwan; lyl3481@gmail.com; 3 Department of Colorectal Surgery, Lo-Hsu Medical Foundation, Lotung Poh-Ai Hospital, Yilan 26546, Taiwan; C059019@mail.pohai.org.tw (M.-C.L.); c843024@mail.pohai.org.tw (Y.-H.Y.); 4 Department of Food Nutrition and Health Biotechnology, College of Medical and Health Science, Asia University, Taichung 41354, Taiwan; 5 Big Data Center, Lo-Hsu Medical Foundation, Lotung Poh-Ai Hospital, Yilan 26546, Taiwan; 6 Division of Radiation Oncology, Lo-Hsu Medical Foundation, Lotung Poh-Ai Hospital, Yilan 26546, Taiwan; 7 Department of Healthcare Administration, College of Medical and Health Science, Asia University, Taichung 41354, Taiwan; 8 Graduate Institute of Business Administration, Fu Jen Catholic University, Taipei 242062, Taiwan; 9 Centers for Regional Anesthesia and Pain Medicine, Wan Fang Hospital, Taipei Medical University, Taipei 11696, Taiwan

**Keywords:** endovascular coil embolization, surgical clipping, aneurysmal subarachnoid hemorrhage, prognostic factors, complications

## Abstract

Scarce evidence is available in Asia for estimating the long-term risk and prognostic factors of major complications such as re-rupture, vasospasm, or re-stroke for patients with aneurysmal subarachnoid hemorrhage (SAH) undergoing endovascular coil embolization or surgical clipping. This is the first head-to-head propensity score-matched study in an Asian population to demonstrate that endovascular coil embolization for aneurysmal SAH treatment is riskier than surgical clipping in terms of re-rupture, vasospasm, or re-stroke. In addition, the independent poor prognostic factors of vasospasm or re-stroke were endovascular coil embolization, male sex, older age (≥65 years; the risk of vasospasm increases with age), hypertension, congestive heart failure, diabetes, previous transient ischemic attack, or stroke in aneurysmal SAH treatment. Background: To estimate the long-term complications and prognostic factors of endovascular coil embolization or surgical clipping for patients with ruptured aneurysmal subarachnoid hemorrhage (SAH). Methods: We selected patients diagnosed with aneurysmal SAH between 1 January 2011 and 31 December 2017. Propensity score matching was performed, and Cox proportional hazards model curves were used to analyze the risk of re-rupture, vasospasm, and re-stroke in patients undergoing the different treatments. Findings: Multivariate Cox regression analysis revealed that the adjusted hazard ratio (aHR) of re-rupture for endovascular coil embolization compared with surgical clipping was 1.36 (95% confidence interval [CI]: 1.17–1.57; *p* < 0.0001). The aHRs of the secondary endpoints of vasospasm and re-stroke (delayed cerebral ischemia) for endovascular coil embolization compared with surgical clipping were 1.14 (1.02–1.27; *p* = 0.0214) and 2.04 (1.83–2.29; *p* < 0.0001), respectively. The independent poor prognostic factors for vasospasm and re-stroke were endovascular coil embolization, male sex, older age (≥65 years; risk increases with age), hypertension, congestive heart failure, diabetes, and previous transient ischemic attack or stroke. Interpretation: Endovascular coil embolization for aneurysmal SAH carries a higher risk than surgical clipping of both short- and long-term complications including re-rupture, vasospasm, and re-stroke.

## 1. Introduction

Aneurysmal subarachnoid hemorrhage (SAH) is a life-threatening event. The primary aim of aneurysmal SAH management is the prevention of re-rupture (re-bleeding) by early repair with surgical clipping or endovascular coiling [1,2,3]. Additional measures are taken to reduce the risk of neurologic and systemic complications, particularly vasospasm and delayed cerebral ischemia (re-stroke) [4]. After aneurysmal SAH, the patient is at substantial risk of early re-rupture (4%–14% in the first 24 h, with maximal risk in the first 2–12 h) [2,5,6,7,8]. Re-rupture is associated with high mortality [2,5,6,7,8]. Aneurysm repair with surgical clipping or endovascular coiling is the only effective treatment to prevent re-rupture [2], but some risk of re-rupture remains even after repair [2,5,6,7,8,9,10,11,12].

Vasospasm and re-stroke are common delayed medical and neurologic complications after aneurysmal SAH repair and substantially increase morbidity and mortality [13,14,15,16]. Several modern neuromonitoring techniques have been developed since 2004 [17,18]. The changes in electrocorticogram, regional cerebral blood flow, and tissue partial pressure of oxygen that occur locally before, during and after the development of delayed cerebral infarction can be recorded by subdural optoelectrodes and intraparenchymal oxygen sensors [18]. Using these technologies and longitudinal neuroimaging, it was shown that the actual delayed cerebral ischemia is a consequence of spreading depolarizations that trigger a severe vasospasm in the microcirculation, which travels together with the neuronal depolarization wave in the gray matter and leads to the delayed infarctions [17]. In these neurovascular events, angiographic (proximal) vasospasm is merely a modifying factor that additionally adversely affects the actual pathophysiological processes in the neurovascular unit. However, the crucial pathophysiological problem is not proximal but distal, and not slow but highly dynamic. Many studies have assessed the risk and prognostic factors of vasospasm or re-stroke in the early phase; however, no long-term follow-up data are available to assess the long-term risk of vasospasm and re-stroke in patients with aneurysmal SAH following treatment with clipping or coiling.

Few studies have described long-term complications such as re-rupture, vasospasm, or re-stroke after standard aneurysmal SAH treatment. No clear head-to-head comparative data exist regarding complications between the two treatment techniques. Therefore, we performed a propensity score-matched study to reduce the selection bias of surgical clipping or endovascular coiling for patients with aneurysmal SAH and to compare the long-term risk of complications (re-rupture, vasospasm, or re-stroke) between the two treatments.

## 2. Patients and Methods

We conducted a population-based cohort study using the Taiwan National Health Insurance (NHI) Research Database (NHIRD). The NHIRD includes all medical claims data on disease diagnoses, procedures, drug prescriptions, demographics, and enrollment profiles of all beneficiaries [19]. We selected patients diagnosed with aneurysmal SAH between 1 January 2011 and 31 December 2017. The follow-up period was from the index date (defined as the date on which clipping or coiling was performed) to 31 December 2018. After adjustment for confounders, a Cox proportional hazards model was established to model the time from the index date to events (re-rupture, vasospasm, or re-stroke) in these patients. Our protocols were reviewed and approved by the Institutional Review Board of Tzu-Chi Medical Foundation (IRB109-015-B). The NHIRD contains detailed treatment-related information regarding the surgical procedures, in-hospital death, surgical clipping, and endovascular coil embolization [20,21,22,23,24,25,26,27,28]. The diagnosis of aneurysmal SAH was confirmed radiologically, and surgical clipping or endovascular coil embolization was performed as treatment. The inclusion criteria were patients ≥ 20 years of age with first aneurysmal SAH diagnosis and early aneurysm repair (within 24–72 h) in patients with good-grade aneurysmal SAH (Hunt and Hess grades I–III). We excluded patients with a history of aneurysmal SAH before the index date, those who did not receive surgical clipping or endovascular coil embolization as treatment, and those who received it >72 h after diagnosis [2]. Sequential intervention, such as surgical clipping for the recurrence of coiled aneurysms, was allowed in the current study because endovascular coil embolization was performed as the first treatment. However, we excluded patients receiving a combination of endovascular and surgical techniques in the same hospitalization because we aimed to compare the individual treatments for risk of complications. Accordingly, we divided the patients into two groups: those receiving endovascular coil embolization (group 1) and those receiving surgical clipping (group 1). Comorbidities were scored using the Charlson comorbidity index (CCI) [29,30]. Diabetes, congestive heart failure, hypertension, renal diseases, stroke, and transient ischemic attack (TIA) were not included in CCI scores to prevent duplicate weighting calculations for risk of postoperative complications. Only comorbidities observed ≥6 months before the index date were included. Comorbid conditions were identified and included according to the diagnostic codes of the International Classification of Diseases, Ninth Revision, Clinical Modification (ICD-9-CM) for the first admission or more than two repeated main diagnosis codes during visits to the outpatient department.

To reduce the effects of potential confounding factors when comparing the risk of complications, propensity score matching (PSM) was performed using a multivariate logistic regression model with the treatment group as the dependent variable and potential confounders as the covariates. Logistic regressions were used for PSM, with the patients receiving endovascular coil embolization as the reference group. Interpretation and the Cox proportional hazards model were dependent on the proportional hazards assumption. Thus, our model assumptions were proportional to the hazards of the different treatments and case-mix variables based on the study by Grambsch [31], who proposed a practical test and an associated graph for examining the critical assumption. A significant *p* value for the global test indicated violation of the proportional hazards assumption for the covariate [31]. Because Schoenfeld residuals assume that the effects of predictor variables are independent of time, a plot of Schoenfeld residuals against time was evaluated to determine whether the effect of the predictor variable changed during follow-up. Both the global test and plot assessment indicated that the proportional hazard assumptions were true for the dataset in the present study. The proportionality of the hazards of the treatment and case-mix variables was assessed. Specifically, we first obtained, by using logistic regression on different treatments, data for the following variables: age, sex, year of diagnosis, location of aneurysm, diabetes, congestive heart failure, hypertension, renal diseases, stroke or TIA, CCI scores, hospital level, hospital area, and income. All patients in the endovascular coil embolization group were matched at a 1:1 ratio with patients in the surgical clipping group through PSM by using global optimization [32]. Multivariate Cox regression analysis was performed to calculate hazard ratios (HRs) to determine whether any of the following variables were significant independent predictors: therapy type, age, sex, year of diagnosis, location of aneurysm, diabetes, congestive heart failure, hypertension, renal diseases, stroke or TIA, CCI score, hospital level, hospital area, and income. The independent predictors were controlled for in the analysis; the primary endpoint was the risk of re-rupture, and the secondary endpoints were the risks of vasospasm and re-stroke in the treatment groups, with surgical clipping serving as the control arm. The definition of vasospasm was a record of vasospasm in the same admission for the first aneurysmal SAH, re-stroke, or re-rupture. Vasospasm was also possibly encountered when receiving treatment for re-rupture or re-stroke after the first aneurysmal SAH. Therefore, the vasospasm might occur and be recorded when admissions of re-rupture or re-stroke undergo treatment.

The risk of complications was estimated using time-dependent Cox proportional hazard curves for vasospasm, re-stroke, and re-rupture in patients receiving different treatments. The hazards were estimated using a proportional subdistribution hazard regression model to overcome the competing risk of death that arises when analyzing the time-to-event data [33]. After we adjusted for confounders, we used the time-dependent Cox proportional hazards method to model the time from the index date to risk of complications. In the multivariate analysis, HRs were adjusted for age, sex, year of diagnosis, aneurysm locations, diabetes, congestive heart failure, hypertension, renal diseases, stroke or TIA previously, CCI score, hospital level, hospital area, and income. All analyses were performed using SAS version 9.3 (SAS Institute, Cary, NC, USA). A two-tailed *p* < 0.05 was considered significant.

## 3. Results

After applying inclusion and exclusion criteria and PSM, 8102 patients (4051 each in the endovascular coil embolization and surgical clipping groups) were considered for analysis; their characteristics are summarized in Supplemental Table S1. Age, sex, year of diagnosis, aneurysm locations, diabetes, congestive heart failure, hypertension, renal diseases, stroke or TIA, CCI score, hospital level, hospital area, and income were similar in the two cohorts (Supplemental Table S1). All standardized differences of each covariate in Supplemental Table S1 were <0.1, indicating a balanced distribution between the two groups [34].

Multivariate Cox regression analysis revealed that treatment was the independent significant prognostic factor of re-rupture (Table 1). Both univariate and multivariate Cox regression analyses indicated that endovascular coil embolization was associated with a higher risk of re-rupture than surgical clipping (adjusted HR [aHR], 1.36; 95% confidence interval [CI]: 1.17–1.57, *p* < 0.0001). After PSM, no significant differences in covariates were observed for the primary endpoint of re-rupture (Table 1), and no residual imbalance or residual confounding bias was observed, indicating that our PSM was satisfactory [35,36].

The aHR (95% CI) of vasospasm for endovascular coil embolization compared with surgical clipping was 1.14 (1.02–1.27), *p* = 0.0214. The aHRs (95% CI) of vasospasm for the age groups of 65–74, 75–84, and 85+ years compared with the age group of 20–64 years were 1.27 (1.11–1.46), 1.40 (1.19–1.66), and 1.07 (1.01–1.53), respectively. The aHRs (95% CI) of vasospasm for men and patients with diabetes, congestive heart failure, hypertension, and stroke or TIA history were 1.22 (1.10–1.37), 1.20 (1.04–1.39), 1.30 (1.06–1.76), 1.17 (1.04–1.32), and 1.27 (1.14–1.43) compared with women and patients without diabetes, congestive heart failure, hypertension, and stroke or TIA history, respectively.

The aHR (95% CI) of re-stroke (delayed cerebral ischemia) for endovascular coil embolization compared with surgical clipping was 2.04 (1.83–2.29), *p* < 0.0001. The aHRs (95% CI) of re-stroke for the age groups of 65–74, 75–84, and 85+ years compared with the age group of 20–64 years were 1.10 (1.06–1.26), 1.21 (1.04–1.20), and 1.22 (1.15–1.52), respectively. The aHRs (95% CI) of re-stroke for men and patients with diabetes, congestive heart failure, hypertension, and stroke or TIA history were 1.06 (1.01–1.19), 1.21 (1.05–1.40), 1.26 (1.11–1.73), 1.14 (1.02–1.28), and 1.27 (1.13–1.42) compared with women and patients without diabetes, congestive heart failure, hypertension, and stroke or TIA history, respectively. Thus, endovascular coil embolization, male sex, older age (≥65 years), diabetes, congestive heart failure, hypertension, and stroke or TIA history were independent poor prognostic factors of both vasospasm and re-stroke (Table 2 and Table 3).

**Figure 1:**
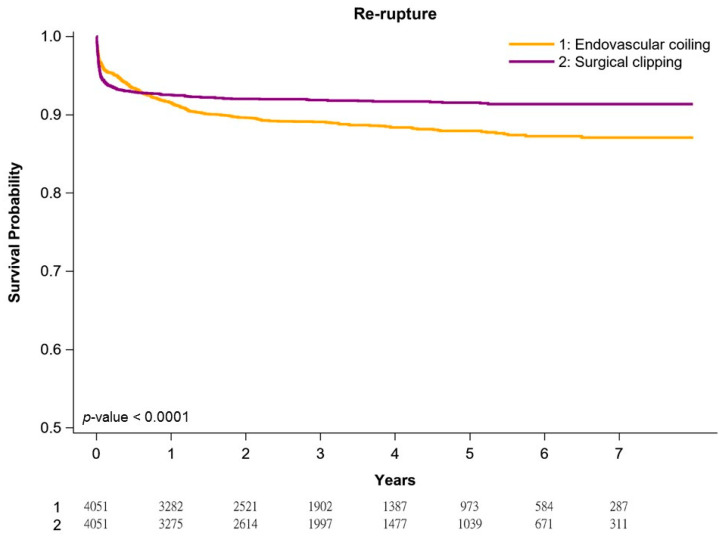
Kaplan–Meier re-rupture-free survival curves of propensity score-matched patients.

**Figure 2:**
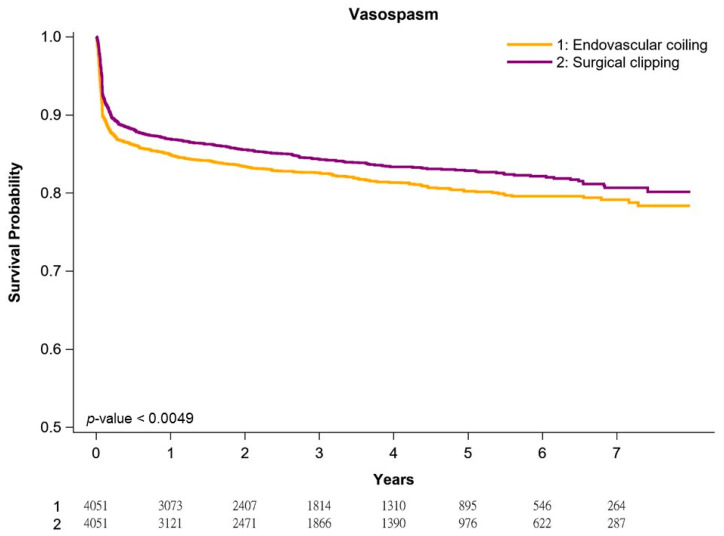
Kaplan–Meier vasospasm-free survival curves of propensity score-matched patients.

**Figure 3:**
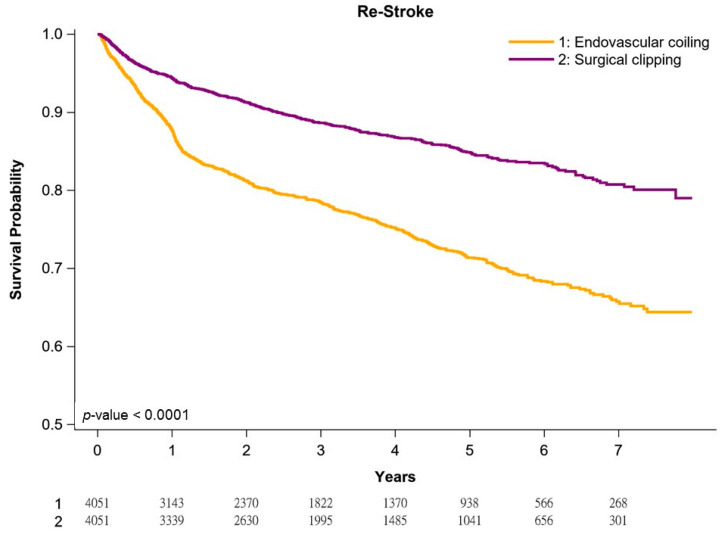
Kaplan–Meier re-stroke-free survival curves of propensity score-matched patients.

Figure 1, Figure 2 and Figure 3 present re-rupture-free, vasospasm-free, and re-stroke-free Kaplan–Meier survival curves in our cohort of patients with aneurysmal SAH. In general, patients undergoing surgical clipping had better survival than those undergoing coil embolization. The 1-year and 5-year re-rupture-free survival rates for surgical clipping and coil embolization were 94.3% and 92.4% and 91.2% and 89.5%, respectively (*p* < 0.0001; Figure 1). The risk of re-rupture was the highest in the first postoperative year and decreased gradually every year, irrespective of the treatment. The 1-year and 5-year vasospasm-free survival rates for surgical clipping and coil embolization were 88.7% and 86.7% and 85.4% and 84.1%, respectively (*p* < 0.0049; Figure 2). The risk of vasospasm was highest after the initial hemorrhage, irrespective of the treatment. The 1-year and 5-year re-stroke-free survival rates for surgical clipping and coil embolization were 95.4% and 88.3% and 86.1% and 74.5%, respectively (*p* < 0.0001; Figure 3). The annual risk of re-stroke continued to be high, irrespective of the treatment.

## 4. Discussion

Surgical clipping and endovascular coiling are the most commonly used treatments for aneurysmal SAH [1,2,3]. In one study, the rate of re-rupture in patients with aneurysmal SAH undergoing endovascular coiling was higher in the first postoperative year than in subsequent years (2.6% versus 1.0%) [33]. Only a few re-rupture events occurred in either treatment group after 1 year, but this was more common in the endovascular coiling group than in the surgical clipping group [33]. In another study, within 8 years, the re-rupture rates were comparable in both groups [37]. Among patients with aneurysmal SAH who reach the hospital alive, early in-hospital mortality is typically caused by the common complications of aneurysmal SAH, namely re-rupture, vasospasm, and re-stroke [38,39,40]. Angiographic (proximal) vasospasm is a risk factor for delayed cerebral ischemia, but is probably not the direct culprit [17,18]. In the early posttreatment phase, vasospasm typically begins no earlier than day 3 after hemorrhage, reaching a peak at days 7 to 8 [9,10,41,42]. Although re-stroke contributes substantially to morbidity and mortality [14,15], no significant association was found between vasospasm and patient outcome. [43]. Other major complications include elevated intracranial pressure related to hydrocephalus or other causes, hyponatremia, seizure, and spreading depolarizations [38,39,40,44]. However, no studies have analyzed the long-term (late-phase) outcomes or prognostic factors of complications such as re-rupture, vasospasm, and re-stroke in patients with aneurysmal SAH receiving coil embolization or surgical clipping, especially in Asia. Understanding the outcomes and prognostic factors of acute and chronic complications is valuable for facilitating shared decision-making by physicians and patients.

In our cohort, no residual imbalance was observed between the two treatment groups (Supplemental Table S1), which explains why no significant factors were noted for the risk of re-rupture (Table 1). Our well-matched PSM design may explain the lack of selection bias in age, sex, year of diagnosis, location of aneurysm, diabetes, congestive heart failure, hypertension, renal diseases, stroke or TIA, CCI score, hospital level, hospital area, and income between the two treatment groups. The crude long-term risks of re-rupture, vasospasm, and re-stroke from 1 January 2011, to 31 December 2017 were 10.5% and 7.8%, 17.2% and 15.2%, and 22.7% and 11.7% for coil embolization and surgical clipping, respectively. More long-term complications of re-rupture, vasospasm, and re-stroke were noted in the endovascular coil embolization group than in the surgical clipping group. Re-rupture of the treated aneurysm occurs in 9%–34% of endovascularly treated aneurysms [45,46,47]. Our results agree with those of studies on re-rupture in patients with aneurysmal SAH receiving endovascular coil embolization or surgical clipping [45,46,47]. Previous studies on vasospasm and re-stroke included only in-hospital data and did not perform long-term follow-ups. Our study is the first to estimate the long-term risk and prognostic factors of complications for patients with aneurysmal SAH receiving endovascular coil embolization or surgical clipping.

The long-term risk of re-rupture between endovascular coil embolization and surgical clipping observed in the present study is compatible with that reported in previous studies [45,46,47]. In the current study, most cases of re-rupture occurred in the first postoperative year irrespective of the treatment; after the first year, re-rupture was rare in the surgical clipping group, but still occurred in the endovascular coil embolization group. Our findings are similar to those of a previous Western study, which indicated few re-rupture events in either treatment group after 1 year, but those that occurred were more common in the coiling group [45]. Our study is the first study from Asia to demonstrate a similar trend of re-rupture between endovascular coil embolization and surgical clipping for patients with aneurysmal SAH; however, the risk of re-rupture in our cohort was higher than that observed in the Western study [45]. Moreover, the prognostic factors of re-rupture were not significant in our study because of a lack of residual imbalance between the two groups after PSM [35,36], meaning that no selection bias was noted for therapeutic choice of endovascular coil embolization or surgical clipping in patients with Hunt and Hess grades I–III aneurysmal SAH in our study.

Consistent with previous studies [9,10,41,42], our data indicated that the risk of vasospasm was the highest after the initial hemorrhage. However, some patients still developed re-rupture or re-stroke combined with vasospasm after the first treatment, and the risk continued to increase even after 7 years of follow up (Figure 2). Our study is the first to evaluate the long-term risk of first vasospasm and sequential vasospasm of the first aneurysmal SAH, re-rupture or re-stroke when receiving treatment in patients with aneurysmal SAH undergoing endovascular coil embolization or surgical clipping. In addition, multivariable Cox regression analysis indicated that the following factors were independent poor prognostic factors of vasospasm: endovascular coil embolization, male sex, older age (≥65 years; the risk of vasospasm increased with age), hypertension, congestive heart failure, diabetes, and previous TIA or stroke (Table 2). Previous studies have also indicated diabetes as the independent poor prognostic factor of vasospasm [48,49]. Several short-term retrospective studies with small sample sizes have revealed conflicting results regarding the association of endovascular coiling with the risk of vasospasm [50,51,52,53,54,55]. Our well-designed PSM study with a large sample size, long-term follow-up, and potentially no selection bias indicated that endovascular coiling is an independent risk factor for vasospasm (Table 2).

Re-stroke is considered when focal neurologic impairment occurs, which was not apparent immediately after aneurysm occlusion and cannot be attributed to other causes after appropriate clinical assessment, brain imaging, and laboratory studies [56]. The most common cause of re-stroke after aneurysmal SAH is assumed to be vasospasm [16]. In our study, unlike re-rupture or vasospasm, the risk of re-stroke was not the highest in the first postoperative year and did not decrease or plateau even after 7 years of follow-up (Figure 3). This could be a regional problem in Taiwan. In the near future, we will investigate in more depth what underlies the unusually high stroke rate in the long term. The first thing to investigate in the future is whether procedures are performed differently in Taiwan than in other countries and whether the differences in these infarcts that occur after years are found only in certain hospitals or in all hospitals. Our findings suggest that Taiwanese patients with aneurysmal SAH receiving treatment should be monitored in the long term for risk of re-stroke, given the crude incidence of 22.7% and 11.7% for endovascular coil embolization and surgical clipping, respectively (Supplemental Table S1). The independent poor prognostic factors for re-stroke were similar to those of vasospasm, which may be because vasospasm is strongly associated with re-stroke [14,15,16]. Our study is the first to estimate the prognostic factors of re-stroke in patients with aneurysmal SAH receiving endovascular coil embolization or surgical clipping after head-to-head PSM and long-term follow-up.

The strengths of this study are its large sample size, long-term follow-up, and the homogeneity of the ruptured intracranial aneurysms population with good-grade aneurysmal SAH, with no heterogeneity in the following covariates: age, sex, year of diagnosis, aneurysm locations, diabetes, congestive heart failure, hypertension, renal diseases, stroke or TIA, CCI score, hospital level, hospital area, and income. Most major covariates were considered in the PSM analysis. This is the first and largest head-to-head PSM study to estimate the long-term major complications of endovascular coil embolization or surgical clipping for patients with aneurysmal SAH. In our findings, endovascular coil embolization had a higher risk than surgical clipping for ruptured intracranial aneurysm. In addition, endovascular coil embolization, male sex, older age (≥65 years), hypertension, congestive heart failure, diabetes, and previous TIA or stroke were independent poor prognostic factors of vasospasm or re-stroke. These findings should be considered in future clinical practice and prospective clinical trials.

This study has some limitations. First, because all patients with aneurysmal SAH were enrolled from an Asian population, our results should be cautiously extrapolated to non-Asian populations in non-areca prevalence areas. Second, the population is aging; instead of vasospasm, there could be the development of atherosclerotic changes in the cerebral arteries, which increase with age in any population. It is possible that such cases were incorrectly assessed as vasospasm because the investigator knew that the patients once had a history of SAH. Therefore, the risk of vasospasm several years after the initial hemorrhage was possibly overestimated. Third, the diagnoses of all comorbid conditions were based on ICD-9-CM codes, which may be entered incorrectly. However, Taiwan’s National Health Insurance Research Administration randomly reviews charts and interviews patients to verify the accuracy of the diagnoses, and hospitals with outlier charges or practices may be audited and subsequently be heavily penalized if malpractice or discrepancies are identified. Nevertheless, a large-scale randomized trial comparing carefully selected patients undergoing suitable treatments is essential to obtain crucial information on population specificity and disease occurrence. Finally, the NHIRD does not contain information regarding dietary habits, socioeconomic status, or body mass index, all of which may be risk factors for mortality. However, considering the magnitude and statistical significance of the observed effects in this study, these limitations are unlikely to affect the results.

## 5. Conclusions

Endovascular coil embolization has a higher risk than surgical clipping for ruptured intracranial aneurysm in terms of acute and long-term complications including re-rupture, vasospasm, and re-stroke. In addition, endovascular coil embolization, male sex, older age (≥65 years; risk increases with age), hypertension, congestive heart failure, diabetes, and previous TIA or stroke were independent poor prognostic factors of vasospasm and re-stroke.

## Figures and Tables

**Table 1:** Cox proportional hazards regression analysis of the risk of re-rupture among the propensity score-matched patients with ruptured subarachnoid aneurysms undergoing surgical clipping or endovascular coiling.

	Univariate			Multivariate		
	HR	(95% CI)	*p* Value	AHR *	(95% CI)	*p* Value
Aneurysm repair modalities						
Surgical clipping	1		<0.0001	1		<0.0001
Endovascular coil embolization	1.34	(1.16–1.55)		1.36	(1.17–1.57)	
Age						
20–64	1		0.2320	1		0.5407
65–74	0.86	(0.71–1.04)		0.86	(0.71–1.05)	
75–84	1.07	(0.43–1.77)		1.04	(0.40–1.17)	
85+	1.03	(0.55–1.93)		1.02	(0.44–1.58)	
Sex						
Female	1		0.5664	1		0.9608
Male	1.04	(0.90–1.21)		1.00	(0.86–1.17)	
Treatment year						
2011–2013	1		0.0913	1		0.1560
2014–2015	1.05	(0.88–1.25)		1.07	(0.90–1.27)	
2016–2017	0.86	(0.72–1.03)		0.89	(0.74–1.07)	
Diabetes	0.79	(0.63–0.99)	0.0451	0.85	(0.67–1.07)	0.1679
Congestive heart failure	0.79	(0.46–1.37)	0.3994	0.86	(0.49–1.51)	0.5984
Hypertension	0.91	(0.79–1.06)	0.2222	1.08	(0.93–1.26)	0.3318
Renal diseases						
No renal diseases	1		0.2274	1		0.5193
Chronic kidney disease	0.90	(0.58–1.41)		1.01	(0.65–1.59)	
End-stage renal disease	1.74	(0.90–3.36)		1.49	(0.75–2.97)	
Stroke or TIA	0.83	(0.71–0.97)	0.0175	0.91	(0.77–1.07)	0.2479
CCI Scores						
0	1		<0.4801	1		0.8913
1	1.04	(0.54–1.27)		1.01	(0.58–1.16)	
2+	1.13	(0.51–1.28)		1.01	(0.56–1.19)	
Hospital level						
Academic centers	1		0.1815	1		0.1847
Nonacademic centers	0.88	(0.73–1.06)		0.88	(0.73–1.06)	
Hospital area						
North	1		<0.5562	1		0.6544
Central	1.08	(0.56–1.54)		1.07	(0.56–1.44)	
South/East	1.05	(0.54–1.58)		1.04	(0.55–1.41)	
Income						
<NTD 18,000	1		0.4338	1		0.5663
NTD 18,000–22,500	1.09	(0.88–1.35)		1.12	(0.90–1.40)	
NTD 22,500–30,000	0.93	(0.74–1.16)		0.96	(0.77–1.20)	
NTD 30,000+	1.08	(0.89–1.31)		1.02	(0.84–1.25)	

CCI, Charlson Comorbidity Index; SD, standard deviation; IQR, interquartile range; NTD, New Taiwan dollar; TIA, transient ischemic attack; CI, confidence interval; AHR, adjusted hazard ratio; HR, hazard ratio. * adjusted for all covariates mentioned in this table.

**Table 2:** Cox proportional hazards regression analysis of the risk of vasospasm among the propensity score-matched patients with ruptured subarachnoid aneurysm undergoing surgical clipping or endovascular coiling.

	Univariate			Multivariate		
	HR	(95% CI)	*p* Value	AHR *	(95% CI)	*p* Value
Aneurysm repair modalities						
Surgical clipping	1		0.0050	1		0.0214
Endovascular coil embolization	1.17	(1.05–1.30)		1.14	(1.02–1.27)	
Age						
20–64	1		<0.0001	1		<0.0001
65–74	1.45	(1.27–1.66)		1.27	(1.11–1.46)	
75–84	1.78	(1.52–2.08)		1.40	(1.19–1.66)	
85+	1.38	(1.08–2.18)		1.07	(1.01–1.53)	
Sex						
Female	1		0.0020	1		0.0004
Male	1.19	(1.07–1.33)		1.22	(1.10–1.37)	
Treatment year						
2011–2013	1		0.3838	1		0.1567
2014–2015	0.96	(0.84–1.10)		0.94	(0.82–1.07)	
2016–2017	0.91	(0.80–1.04)		0.87	(0.75–1.00)	
Diabetes	1.47	(1.28–1.68)	<0.0001	1.20	(1.04–1.39)	0.0141
Congestive heart failure	1.69	(1.16–2.27)	0.0005	1.30	(1.06–1.76)	0.0297
Hypertension	1.35	(1.21–1.51)	<0.0001	1.17	(1.04–1.32)	0.0089
Renal diseases						
No renal diseases	1		0.0364	1		0.2182
Chronic kidney disease	1.43	(1.09–1.89)		1.09	(0.82–1.44)	
End-stage renal disease	1.09	(0.58–2.02)		0.60	(0.32–1.11)	
Stroke or TIA	1.31	(1.18–1.46)	<0.0001	1.27	(1.14–1.43)	<0.0001
CCI Scores						
0	1		0.3241	1		0.6873
1	0.91	(0.78–1.07)		1.01	(0.68–1.05)	
2+	1.30	(0.90–1.54)		1.02	(0.85–1.22)	
Hospital level						
Academic centers	1		0.7209	1		0.1738
Nonacademic centers	0.98	(0.85–1.12)		0.91	(0.79–1.04)	
Hospital area						
North	1		0.4004	1		0.0570
Central	0.90	(0.78–1.05)		0.84	(0.72–1.07)	
South/East	0.96	(0.85–1.10)		0.96	(0.84–1.10)	
Income						
<NTD 18,000	1		0.0006	1		0.1133
NTD 18,000–22,500	1.00	(0.86–1.17)		1.05	(0.90–1.23)	
NTD 22,500–30,000	0.97	(0.82–1.13)		1.04	(0.88–1.22)	
NTD 30,000+	0.77	(0.66–0.89)		1.04	(0.73–1.08)	

CCI, Charlson Comorbidity Index; SD, standard deviation; IQR, interquartile range; NTD, New Taiwan dollar; TIA, transient ischemic attack; CI, confidence interval; AHR, adjusted hazard ratio; HR, hazard ratio. * adjusted for all covariates mentioned in Table 1.

**Table 3:** Cox proportional hazards regression analysis of the risk of re-stroke among propensity score-matched patients with ruptured subarachnoid aneurysm undergoing surgical clipping or endovascular coiling.

	Univariate			Multivariate		
	HR	(95% CI)	*p* Value	AHR *	(95% CI)	*p* Value
Aneurysm repair modalities						
Surgical clipping	1		<0.0001	1		<0.0001
Endovascular coil embolization	2.08	(1.86–2.32)		2.04	(1.83–2.29)	
Age						
20–64	1		<0.0001	1		0.0135
65–74	1.30	(1.14–1.48)		1.10	(1.06–1.26)	
75–84	1.50	(1.27–1.77)		1.21	(1.04–1.20)	
85+	1.22	(1.12–2.06)		1.22	(1.15–1.52)	
Sex						
Female	1		0.1537	1		0.0416
Male	1.08	(0.97–1.21)		1.06	(1.01–1.19)	
Treatment year						
2011–2013	1		<0.7911	1		<0.8801
2014–2015	0.91	(0.97–2.66)		0.95	(0.70–2.23)	
2016–2017	0.78	(0.84–4.41)		0.91	(0.84–3.34)	
Diabetes	1.45	(1.26–1.66)	<0.0001	1.21	(1.05–1.40)	0.0099
Congestive heart failure	1.75	(1.29–2.36)	0.0003	1.26	(1.11–1.73)	0.0151
Hypertension	1.23	(1.11–1.37)	<0.0001	1.14	(1.02–1.28)	0.0239
Renal diseases						
No renal diseases	1		0.0053	1		0.7824
Chronic kidney disease	1.35	(1.01–1.80)		0.94	(0.70–1.28)	
End-stage renal disease	2.00	(1.18–3.38)		0.84	(0.49–1.46)	
Stroke or TIA	1.26	(1.13–1.40)	<0.0001	1.27	(1.13–1.42)	<0.0001
CCI Scores						
0	1		0.5607	1		0.3244
1	0.78	(0.67–1.90)		0.84	(0.72–1.48)	
2+	0.91	(0.78–1.07)		0.79	(0.67–1.44)	
Hospital level						
Medical center	1		0.2219	1		0.6973
others	1.08	(0.95–1.23)		0.97	(0.85–1.11)	
Hospital area						
North	1		0.5405	1		<0.6611
Central	1.06	(0.66–1.88)		1.03	(0.58–1.78)	
South/East	1.08	(0.60–1.78)		1.04	(0.56–1.73)	
Income						
<NTD 18,000	1		<0.0001	1		0.2340
NTD 18,000–22,500	0.80	(0.68–0.94)		1.05	(0.89–1.25)	
NTD 22,500–30,000	1.35	(1.16–1.57)		1.17	(1.00–1.37)	
NTD 30,000+	0.97	(0.84–1.13)		1.04	(0.89–1.20)	

CCI, Charlson Comorbidity Index; SD, standard deviation; IQR, interquartile range; NTD, New Taiwan dollar; TIA, transient ischemic attack; CI, confidence interval; AHR, adjusted hazard ratio; HR, hazard ratio. * adjusted for all covariates mentioned in Table 1.

## Data Availability

The datasets supporting the study conclusions are included in this manuscript and its Supplementary Files.

## Supplementary Materials

The following are available online at https://www.mdpi.com/article/10.3390/jpm11111209/s1, Table S1: Demographic and clinical parameters of Propensity Score Matching patients with rupture intracranial aneurysm.
