# Supplementary material for: Risk of Re-Rupture, Vasospasm, or Re-Stroke after Clipping or Coiling of Ruptured Intracranial Aneurysms: Long-Term Follow-Up with a Propensity Score-Matched, Population-Based Cohort Study
Source: J Pers Med. 2021 Nov 16;11(11):1209. doi: 10.3390/jpm11111209 (PMC8622401; doi:10.3390/jpm11111209)
Supplement: Supplementary file 1 [file jpm-11-01209-s001.zip › jpm-1416430-supplementary.pdf]

Supplemental Table S1. Clinicodemographic parameters of propensity score–matched patients with ruptured subarachnoid aneurysm.

		Endovascular coil embolization N=4051		Surgical clipping N=4051		Standardized difference
		n	(%)	n	(%)	
Age	Mean (SD)	58·0	(13·7)	57·5	(13·1)	0·035
	20–64	2716	(67·0)	2839	(70·1)	0·028
	65–74	791	(19·5)	746	(18·4)	0·047
	75–84	474	(11·7)	415	(10·2)	0·039
	85+	70	(1·7)	51	(1·3)	0·035
Sex	Male	1479	(36·5)	1432	(35·3)	0·024
	Female	2572	(63·5)	2619	(64·7)	
Treatment year	2011–2013	1412	(34·9)	1481	(36·6)	0·012
	2014–2015	1172	(28·9)	1194	(29·5)	0·047
	2016–2017	1467	(36·2)	1376	(34·0)	0·042
Location of aneurysm	ACA	1860	(45·9)	1860	(45·9)	0·000
	ICA	376	(9·3)	376	(9·3)	0·000
	MCA	1372	(33·9)	1372	(33·9)	0·000
	VBA	302	(7·5)	302	(7·5)	0·000
	PCA	141	(3·5)	141	(3·5)	0·000
Diabetes	No	3437	(84·8)	3497	(86·3)	0·040
	Yes	614	(15·2)	554	(13·7)	

		Endovascular coil embolization N=4051		Surgical clipping N=4051		Standardized difference
		n	(%)	n	(%)	
Congestive heart failure	No	3945	(97·4)	3962	(97·8)	0·027
	Yes	106	(2·6)	89	(2·2)	
Hypertension	No	1895	(46·8)	1983	(49·0)	0·043
	Yes	2156	(53·2)	2068	(51·0)	
Renal diseases	End-stage renal disease	45	(1·1)	32	(0·8)	0·038
	Chronic kidney disease	138	(3·4)	118	(2·9)	0·028
	No renal diseases	3868	(95·5)	3901	(96·3)	0·041
Stroke or TIA	No	2440	(60·2)	2616	(64·6)	0·090
	Yes	1611	(39·8)	1435	(35·4)	
CCI Scores	0	628	(15·5)	683	(16·9)	0·013
	1	2304	(56·9)	2353	(58·1)	0·024
	2+	1119	(27·6)	1015	(25·1)	0·058
Hospital levels	Academic centers	3178	(78·4)	3198	(78·9)	0·012
	Nonacademic centers	873	(21·6)	853	(21·1)	
Hospital areas	North	2329	(57·5)	2172	(53·6)	0·055
	Central	735	(18·1)	843	(20·8)	0·067
	South	871	(21·5)	915	(22·6)	0·026
	East	116	(2·9)	121	(3·0)	0·007

		Endovascular coil embolization N=4051		Surgical clipping N=4051		Standardized difference
		n	(%)	n	(%)	
Income levels	<NT$ 18,000	924	(22·8)	872	(21·5)	0·026
	NT$ 18,000–22,500	842	(20·8)	903	(22·3)	0·037
	NT$ 22,500–30,000	915	(22·6)	908	(22·4)	0·004
	NT$ 30,000+	1370	(33·8)	1368	(33·8)	0·001

		Endovascular coil embolization N=4051		Surgical clipping N=4051		***P* Value**
		N	(%)	N	(%)	
Vasospasm		696	(17·2)	615	(15·2)	0·0143
Re-stroke		921	(22·7)	473	(11·7)	<0·0001
Re-rupture		424	(10·5)	317	(7·8)	<0·0001

CCI, Charlson Comorbidity Index; SD, standard deviation; IQR, interquartile range; NT$, New Taiwan dollar; TIA, transient ischemic attack; ACA, anterior cerebral artery; ICA, internal carotid artery; MCA, middle cerebral artery; VBA, vertebrobasilar artery; PCA, posterior cerebral artery.
